# 5-ALA induced PpIX fluorescence spectroscopy in neurosurgery: a review

**DOI:** 10.3389/fnins.2024.1310282

**Published:** 2024-01-29

**Authors:** A. Gautheron, J. D. Bernstock, T. Picart, J. Guyotat, P. A. Valdés, B. Montcel

**Affiliations:** ^1^Université Jean Monnet Saint-Etienne, CNRS, Institut d Optique Graduate School, Laboratoire Hubert Curien UMR 5516, Saint-Étienne, France; ^2^Univ Lyon, INSA-Lyon, Université Claude Bernard Lyon 1, UJM-Saint Etienne, CNRS, Inserm, CREATIS UMR 5220, U1294, Lyon, France; ^3^Department of Neurosurgery, Brigham and Women’s Hospital and Harvard Medical School, Boston, MA, United States; ^4^David H. Koch Institute for Integrative Cancer Research, Massachusetts Institute of Technology, Cambridge, MA, United States; ^5^Department of Neurosurgical Oncology and Vascular Neurosurgery, Pierre Wertheimer Neurological and Neurosurgical Hospital, Hospices Civils de Lyon, Lyon, France; ^6^Université Lyon 1, INSERM 1052, CNRS 5286, Lyon, France; ^7^Department of Neurosurgery, University of Texas Medical Branch, Galveston, TX, United States; ^8^Department of Neurobiology, University of Texas Medical Branch, Galveston, TX, United States; ^9^Department of Electrical and Computer Engineering, Rice University, Houston, TX, United States

**Keywords:** fluorescence spectroscopy, 5-aminolevulinic acid (5-ALA), protoporphyrin IX (PpIX), fluorescence guided surgery, brain tumors, neurooncology, image guidance, neurosurgery

## Abstract

The review begins with an overview of the fundamental principles/physics underlying light, fluorescence, and other light-matter interactions in biological tissues. It then focuses on 5-aminolevulinic acid (5-ALA)-induced protoporphyrin IX (PpIX) fluorescence spectroscopy methods used in neurosurgery (e.g., intensity, time-resolved) and in so doing, describe their specific features (e.g., hardware requirements, main processing methods) as well as their strengths and limitations. Finally, we review current clinical applications and future directions of 5-ALA-induced protoporphyrin IX (PpIX) fluorescence spectroscopy in neurosurgery.

## 1 Introduction

Clinical practice in neurosurgery has evolved dramatically over the last 20 years. Tumor surgery in particular has been advanced by the advent of fluorescence-guided surgery (FGS; McCracken et al., 2022). FGS is a powerful surgical adjunct and has improved the rates of complete resection(s) of brain tumors, specifically in high-grade gliomas (HGG; Stummer et al., 1998b; Hadjipanayis et al., 2015). However, one major limitation related to current practices in FGS in neurosurgery using conventional surgical microscopes modified for fluorescence imaging (fluorescence microscopy) is the resultant sensitivity of tumor signals. Tumor cells in the more infiltrative regions of gliomas do not demonstrate visible fluorescence when conventional microscopy visualization is employed, which leads to residual tumor tissue going undetected (Stummer et al., 2000; Floeth and Stummer, 2005; Utsuki et al., 2006; Ishihara et al., 2007; Hefti et al., 2008; Ruge and Liu, 2009; Widhalm et al., 2010; Ewelt et al., 2011; Floeth et al., 2011; Sanai et al., 2011b; Valdés et al., 2011b; Montcel et al., 2013; Beez et al., 2014; Marbacher et al., 2014). To improve detection within these infiltrative regions, acquisition of the complete fluorescence spectrum of fluorescent agents has been proposed, given that spectrally-resolved acquisition would identify the “optical fingerprint” of fluorescent biomarkers, providing more sensitive and specific information than the color images obtained with conventional fluorescence microscopy.

This review begins by describing the key fundamental principles of light, including relevant elements of fluorescence and other pertinent light-matter interactions in biological tissues. 5-aminolevulinic acid (5-ALA)-induced protoporphyrin IX (PpIX) fluorescence spectroscopy methods used in neurosurgery are then examined, along with their specific hardware requirements/main processing methods and associated limitations. The final section looks at both current and future clinical applications of 5-ALA-induced PpIX fluorescence spectroscopy in neurosurgery.

## 2 Fundamentals of light, fluorescence, and light-matter interactions

**Figure 1:**
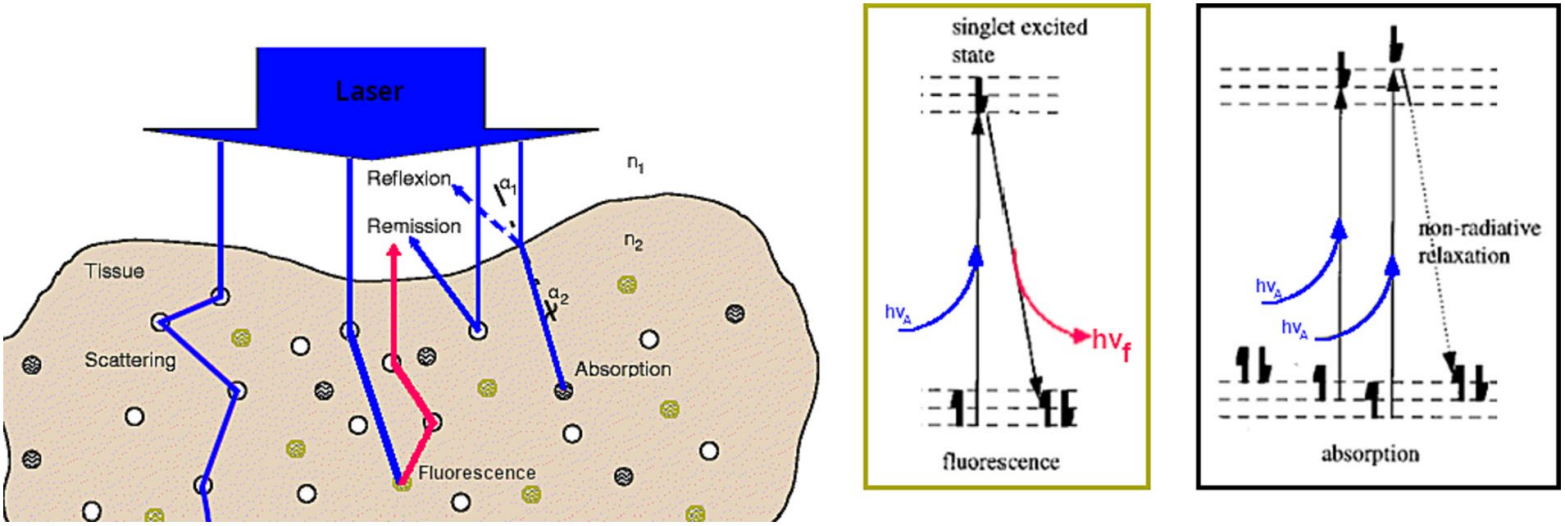
Schematic describing light-biological tissue interactions (absorption, scattering and fluorescence) at the tissue (left). and at the molecular level (right). Adapted from Richards-Kortum and Sevick-Muraca (1996) and Steiner (2011).

When light interacts with biological tissue, light absorption and scattering occur whatever of the light source and the tissue type (Tuchin, 1994, 2015b; Figure 1). Other light phenomena such as luminescence, phosphorescence, fluorescence, and/or Raman scattering can occur depending on conditions/context (Tuchin, 1994, 2015b). Absorption of light occurs when the energy of an incident photon matches an electronic or vibrational transition of a chromophore, which, in medical biology, is a molecules capacity of absorbing radiation; this energy is ultimately dissipated in tissue as heat (Arridge et al., 1992; Tuchin, 1993, 2015c). At any given wavelength, this absorption phenomenon is described by the wavelength-dependent linear absorption coefficient μ_a_(λ) (Cheong et al., 1990; Taroni et al., 2003; Jacques, 2013a). When considering biological tissues, the main absorbers in the visible and near-infrared (NIR) wavelength range are oxy- and deoxy-hemoglobin and water. Thus, higher concentrations of blood and water result in greater absorption in the visible and NIR ranges, respectively (Serebrennikova et al., 2008).

Scattering of light is due to changes or fluctuations in the refractive index; the refractive index of a medium is defined as the ratio between the speed of light in a vacuum and the speed of light in the medium it passes through (Hébert, 2022). Please note that, as for absorption, the wavelength-dependent scattering coefficient μ_s_(λ) is defined for each individual tissue (Jacques, 2013a,b). In biologic tissues, cells/organelles are primarily responsible for such scattering (Mourant et al., 1998). Thus, a higher tissue cellularity with fixed absorption would result in a higher scattering coefficient. Moreover, light scattering implies a change in the propagation direction of light. In many cases, scattered light is assumed to be isotropically distributed over all angles. However, an angular dependence of light scattering, called anisotropy, exists, especially in biological medias. To model it, light propagation models use *phase functions* to translate this phenomenon of angular dependency of elastic scattering. Different phase functions exists depending on the size of the scattering particles [Mie scattering (Mie, 1908; Henyey and Greenstein, 1941), Rayleigh scattering (Strutt, 1871; Rayleigh, 1881)]. Of note, light scattering within tissues is generally not isotropic and as such an anisotropy factor *g* is introduced, which varies from −1 for predominantly backward scattering, to 1 for predominantly forward scattering, with 0 representing isotropic scatter. For biological tissues, the anisotropy factor is often close to one (Jacques, 2013a,b).

Due to constraints related to biological tissues, we only have access to the reflectance measurement to determine absorption and scattering. The term *reflectance* denotes any ratio of reflected flux to incident flux relative to the same surface element (Hébert, 2022), where the flux is the energy radiated through a surface per unit time. This quantity is dimensionless and depends on wavelength, direction, polarization, and position on the surface. As most instruments contain one detector which captures the reflected flux, the incident flux cannot be measured directly. The incident flux is thus measured indirectly by using a perfect white diffuser able to reflect the incident light uniformly over the hemisphere without absorbing it. In practice, white standards approaching these properties are made of pressed barium sulfate or PTFE (e.g., Spectralon®; CIE, 1986). The flux captured by the detector is therefore proportional to the incident flux. As they are calibrated in terms of the perfectly reflecting diffuser, it is then possible to access the incident flux (CIE, 1979). Then, the ratio of the flux measured from the object to the incident flux is called reflectance. In the field of biological imaging, the reflectance varies according to the optical properties, in the sense that the reflectance will be very low especially if the medium is very absorbent or if the scattering is strong and the measurement area is small compared to the scattering halo (Cui and Ostrander, 1992).

Fluorescence is a phenomenon unique to certain molecules which we call fluorophores or fluorescent agents (Tuchin, 2015a). Fluorescence occurs when the fluorophore absorbs an incident photon’s energy, which causes its electrons to move from a ground state to an excited state. After a step of internal reconversion of the absorbed energy, the return to the equilibrium of the electrons return to their original equilibrium state by either by emission of lower energy photons, i.e., photons of longer wavelength than the one initially absorbed photons or a non-radiative release of energy. Further, the energy required to excite a molecule depends on its energy levels, which are unique to the chemical composition of each molecule. The absorption of a photon at the frequency ν by a molecule is governed by the relationship E = hν where E is the energy required, h the Planck constant, and ν the frequency of the incident photon. Each fluorophore has a wavelength-dependent absorption spectrum that is unique to it, which enables identification of the optimal excitation wavelengths. For some fluorescent molecules, including porphyrins, the Soret band is defined as the ultraviolet (UV) wavelength where the absorption is at its maximum, i.e., the maximum of the absorption spectrum.

Fluorophores have different properties that are used to characterize them. The quantum yield η of a fluorescent molecule is defined as the number of photons emitted over the number of photons absorbed. The spectral extinction coefficient reveals the probability of absorption of photons by a molecule according to their wavelength. The fluorescence spectrum emitted by a molecule represents the number of photons emitted depending on their wavelength and its shape is often independent of the excitation source. This spectrum is specific to a molecule and can be described as the “optical fingerprint” of that molecule. Fluorescence lifetime is an intrinsic property of a fluorophore, which corresponds to the time spent by the fluorophore in the excited state before emitting a photon and returning to the ground state. For example, for common fluorescent dyes used in neurosurgery, the lifetime is between 4.0 nanoseconds for fluorescein and up to 15.0 ns for PpIX (Berezin and Achilefu, 2010). Fluorescence lifetime measurements are denoted as the fluorescence intensity as a function of time at a specified excitation-emission wavelength pair (Ramanujam, 2000). Finally, whether studying the fluorescence lifetime or the fluorescence spectrum of a tissue, it can be considered as the sum of the fluorescence lifetimes, resp. fluorescence spectra, of each of its components, weighted by their properties (quantum yield, extinction coefficient, concentration, absorption, etc.). Thus, the study of the fluorescence lifetime or spectra of biological tissues allows us to identify their properties (Patterson and Pogue, 1994).

## 3 Methods/techniques of fluorescence spectroscopy

FGS as an adjunct in neurosurgery has garnered significant enthusiasm given the positive results from a phase III clinical trial that explored the use of 5-ALA induced PpIX fluorescence in HGG. This work demonstrated an ~doubling in the rates of gross total resections (GTR) for high-grade gliomas when 5-ALA was employed (Stummer et al., 2006).

**Figure 2:**
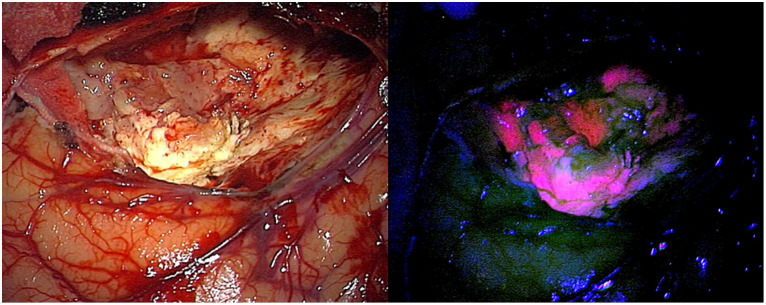
Healthy brain and glioblastoma image through microscope under white light (left) and blue light with re-emission after 440 nm (right).

Exogenous administration of 5-ALA leads to significant accumulation of PpIX in a variety of tumor tissues including in HGG, meningiomas, metastases, and in some instances, low-grade gliomas (LGG; Hadjipanayis et al., 2015b; Valdés et al., 2019; Bernstock et al., 2022; McCracken et al., 2022). Typically, patients are given a dose of 5-ALA (20 mg/kg) ~2–4 h prior to surgery, which leads to PpIX accumulating in tumor tissues. Then, using a surgical microscope equipped with 405 nm excitation light and filters to collect the emitted red-pink fluorescence via a camera or through the surgeon’s oculars (Stummer et al., 1998a; Valdés et al., 2016, 2019; McCracken et al., 2022) one can perform FGS. Specifically, the surgeon switches between white light illumination mode for conventional imaging of the brain parenchyma and surgical debris (Figure 2) to fluorescence mode to enable enhanced visualization of red-pink, fluorescent tumor tissue, or non-fluorescent tissue (e.g., non-contrast enhancing tissue; Figure 2). 5-ALA-PpIX FGS provides high tumor-to-non-tumor tissue contrast with visualization in real time unlike many of the other currently employed surgical technologies: for example, neuronavigation suffers from brain shift and often becomes inaccurate during the course of surgery; intraoperative magnetic resonance imaging (iMRI) represents a singular point in time and is also prone to brain shift, can lead to surgical delays, and increased costs, while the poor tumor-to-normal contrast provided by intraoperative ultrasound remains suboptimal in guiding resections (Valdés et al., 2010, 2016; McCracken et al., 2022). It is prudent to note that 5-ALA-PpIX fluorescence also provides a strong intraoperative correlate of critical imaging features (i.e., the PpIX red-pink fluorescence) when compared to preoperative imaging features (i.e., contrast enhancement on MRI); such relationships are in fact confirmed on resultant histopathology (i.e., high positive predictive value of PpIX fluorescence for tumor; Stummer et al., 2000, 2006; Roberts et al., 2011; Valdés et al., 2011a, 2012b). In addition, 5-ALA-PpIX integrates easily into standard neurosurgical workflows, with oral administration 2–4 h prior to the induction of anesthesia. Such timing ensures the preservation of fluorescence over the course of a typical glioma surgery with a signal that can be visualized with conventional systems (i.e., surgical microscopes, exoscopes and/or loupes; Pogue et al., 2010; Suero Molina et al., 2021).

It is also prudent to note that 5-ALA-PpIX has a significant history in the photodynamic literature for the treatment of non-central nervous system cancers, e.g., skin cancers (Stummer et al., 1998a; Pogue et al., 2010). However, the groundbreaking work by Stummer et al. in 52 patients with high-grade gliomas first demonstrated its clinical utility for demarcating contrast-enhancing high grade brain tumors (Stummer et al., 2000). This first in human study subsequently lead to the landmark phase III randomized controlled trial in 270 patients (i.e., conventional white light guided resection vs. 5-ALA-PpIX FGS; Stummer et al., 2006). This study showed that use of 5-ALA-PpIX FGS lead to an almost doubling in the rate of GTR in high grade glioma with 65% in the FGS group as compared to 36% in the white light (non-FGS) group having achieved GTR. Furthermore, more recently a randomized phase III multicenter French collaborative study (Picart et al., 2023) confirmed that FGS significantly improved the extent of resection in glioblastoma (GBM), and thus, increased the overall rates of GTR of contrast enhancing tumor in patients managed according to the current standards of care (i.e., microsurgical resection, neuronavigation); when reexamined, GTR remained a significant independent predictor of progression free survival and overall survival. Finally, a prospective multicenter controlled parallel group trial evaluated the use of 5-ALA FGS compared to intraoperative MRI (iMRI) in 314 GBM patients, and found that complete resection rates, overall survival and progression free survival were not significantly different between the 5-ALA and iMRI groups (Roder et al., 2023). However, they did find that incision to suture times were significantly longer for iMRI vs. FGS group (316 vs. 215 min, respectively; *p* < 0.001). This study shows that 5-ALA FGS is not-inferior to iMRI in terms of extent of resection, OS, and PFS, but might have an economic impact for GBM given the high costs and extra surgical time associated with iMRI use.

However, despite the clinical success and adoption of PpIX fluorescence as a standard of care for the resection of high-grade gliomas in FGS, limitations have been demonstrated in terms of the accuracy of tumor and/or margin detection using PpIX FGS. Thus, fluorescence spectroscopy has been developed to address the limitations of conventional fluorescence microscopy for identifying tumor margins (Toms et al., 2005; Stummer et al., 2006; Bravo et al., 2017).

This review focuses on 5-ALA-PpIX fluorescence, however, it is important to note the use of autofluorescence (AF) spectroscopy for brain diagnostics (Richards-Kortum and Sevick-Muraca, 1996). AF is a widespread phenomenon due to the intrinsic presence of many biomolecules in living organisms such as nicotinamide adenine dinucleotide hydrate (NADH), flavin adenine dinucleotide (FAD), flavin mononucleotide (FMN), collagen, elastin, and porphyrins. These biomolecules act as endogenous fluorophores, closely linked to the morphological or functional properties of living systems, which influence their AF emission characteristics. This makes AF an extremely powerful resource for directly monitoring biological systems.

The applications of AF spectroscopy in neurosurgery are mentioned in Pogue et al. (2010) as well as in an abundant literature of which some articles, such as Bottiroli et al. (1998) and Croce et al. (2003), present AF spectroscopy as an intra-operative aid to the delineation of infiltrated areas in brain tumors. The scientific focus on AF spectroscopy, as well as the links between endogenous biomolecules and pathologies from a contrast point of view, are comprehensively described in the following reviews (Croce and Bottiroli, 2014; Vasefi et al., 2017).

In order to detect tumor margins more accurately, research groups, including ours, have developed handheld intraoperative spectroscopy tools for acquiring fluorescence and/or white light spectra (Gardner et al., 1996; Richards-Kortum and Sevick-Muraca, 1996; Gebhart et al., 2006; Utsuki et al., 2006, 2007; Butte et al., 2010, 2011; Haj-Hosseini et al., 2010; Richter et al., 2011; Butte and Mamelak, 2012; Valdés et al., 2012c, 2015; Montcel et al., 2013; Kittle et al., 2016; Alston et al., 2019). All these single point spectroscopic tools can be clustered into two major categories, (a) steady-state intensity fluorescence spectroscopy and (b) time-resolved fluorescence spectroscopy. Here, we provide a summary of each of these forms of spectroscopy implemented in FGS given the distinct technical requirements in terms of hardware and software as well as an overview of clinical implementations of both of these forms of spectroscopy in FGS for brain tumor applications.

### 3.1 Fluorescence intensity spectroscopy

Traditionally, spectroscopic probes measure the fluorescence intensity because they are connected to a spectrometer which captures the number of emitted photons in a wavelength dependent manner. Basic fluorescence intensity systems are compact as they consist of three main components: (1) optical fibers connected to (2) light source(s) to illuminate the tissue and also connected to (3) a spectrometer to collect the fluorescence emissions and/or reflected light in a spectrally-resolved manner (Stummer et al., 1998a; Utsuki et al., 2006; Haj-Hosseini et al., 2010; Richter et al., 2011).

**Figure 3:**
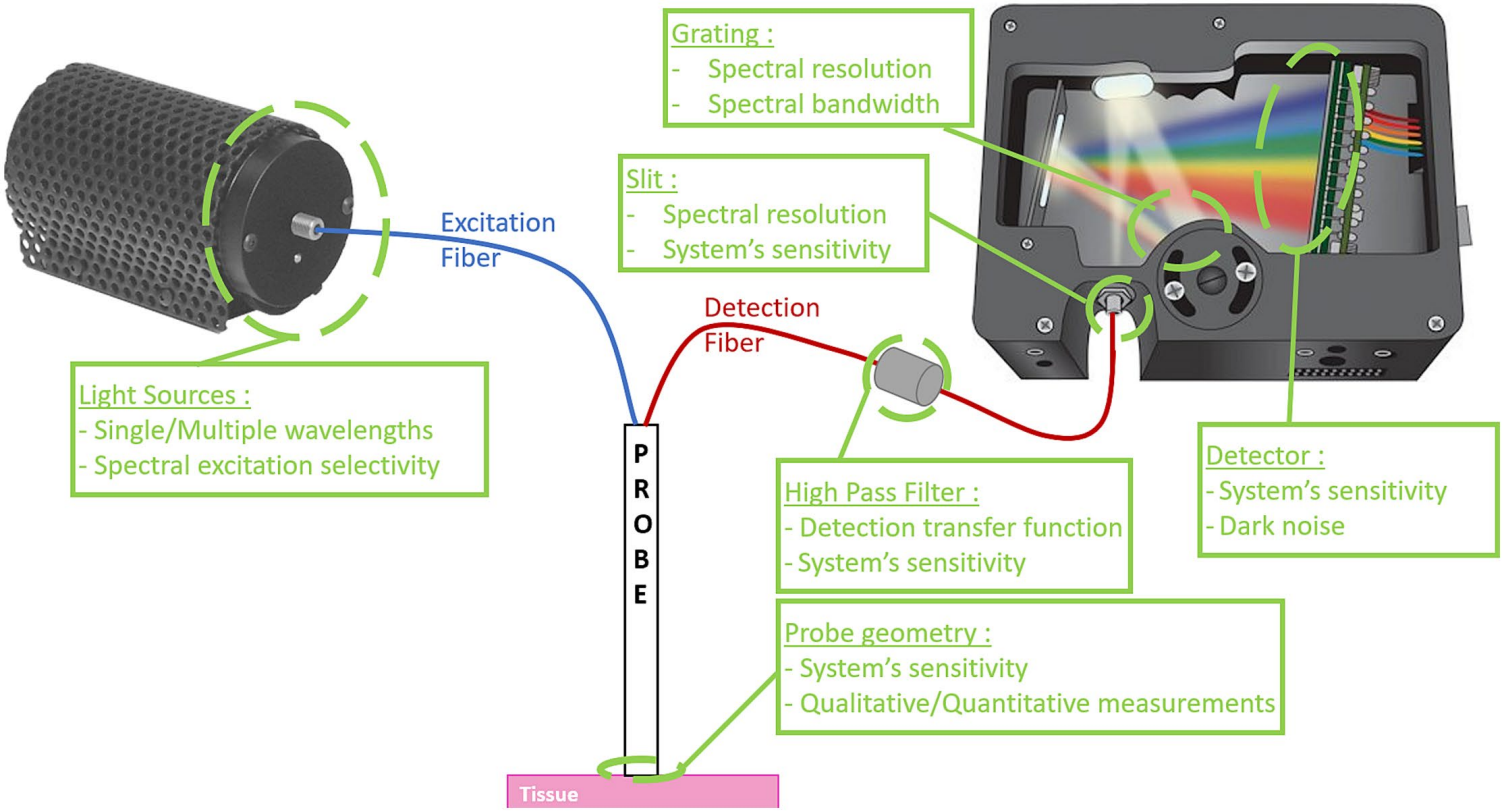
Schematic diagram of a fluorescence intensity spectroscopy system. On the left, the excitation source(s), followed in blue by the excitation fiber(s) that carry(ies) the signal to the probe tip. In red, the detection fiber(s) that guide(s) the collected signal to the spectrometer. System characteristics influenced by the various elements are described in green. Some images are used with the courtesy from Ocean Optics.

Several components of fluorescence intensity spectroscopy systems influence the characteristics, including sensitivity, bandwidth and spectral resolution. The characteristics of some experimental setups have been grouped in Table 1. In addition, Figure 3 schematically summarizes the various elements of the experimental set-up and their impact on the characteristics.

**Table 1:** Comparison of well-known fluorescence intensity spectroscopy systems.

	Haj-Hosseini et al. (2010)	Stummer et al. (2014) and Stummer (2015)	Alston et al. (2019)	Dartmouth-Toronto Team (Kim et al., 2010; Valdés et al., 2011b, 2012c)
Excitation source(s)	1 Laser Diode @ 405 nm	D-Light System (broadband)	3 LED @ 375 nm, 405 nm, 420 nm	LED @ 405 nm
Spectral resolution	3 nm	Unknown	4 nm	0.1–10 nm
Spectral bandwidth	240–850 nm	Unknown	220–1,100 nm	450–850 nm
Probe geometry	Concentric:	Concentric:	Concentric:	Linear:
	one central excitation fiber nine detection fibers	one central excitation fiber six detection fibers	one central detection fiber seven excitation fibers	array of four optical fibers
Pros	Higher sensitivity (large number of photons captured by the detection fibers) Greater uniformity of explored field		Multi spectral excitation Higher sensitivity Greater uniformity of explored field	Able to quantify fluorescence
Cons	Performs only qualitative intensity fluorescence spectroscopy			Less signal detected

The point probes featured in existing systems have different geometries, depending on system requirements. In Haj-Hosseini et al. (2010), Stummer et al. (2014), and Stummer (2015), to optimize the amount of signal detected, the probe geometry is concentric: a single central excitation fiber with a large core, and a ring of smaller-core fibers for localized detection around the excitation fiber. In Alston et al. (2019), the probe geometry is also concentric but the excitation fibers are around the central detection fiber. A small volume of glass is present between the end of the optical fibers and the tissue, in such a way that the illumination zone and the detection zone overlap. In all previous cases, the probe’s geometry enables a greater uniformity of the field, with a high sensitivity due to a huge quantity of collected photons. However, these probes allow only qualitative fluorescence measurements. To reach the quantitative objective, in Dartmouth’s team (Kim et al., 2010; Valdés et al., 2011c, 2012c), the point probe is made up of a linear array of 4 small core diameter fibers. As such, the probe has been designed to measure both fluorescence and white light diffuse reflectance in a broad wavelength range. This addition of diffuse reflectance spectroscopy (DRS) measurement implies the construction of more complex experimental systems (i.e., illumination and collection fibers at known fixed distances), a more complex acquisition protocol (i.e., sequence with multiple illumination and detection events, calibration to known standards), and use of compatible light transport models for estimation of tissue optical properties and correction of the fluorescence.

Common systems use spectrometers for the spectrally resolved detection part. Depending on the type of spectrometer (UV/VIS, VIS/NIR or even NIR-II), the spectral measurement bandwidth changes. In addition, the spectrometer contains a grating whose characteristics influence detection sensitivity, which is also modified by the sensitivity of the sensor [charge-coupled device (CCD)]. Finally, the size of the slit at the spectrometer entrance also influences the spectral resolution of the system. The spectral resolution of systems in the literature is a few nanometers. Finally, high-pass filters are used between the probe and the spectrometer to remove the excitation signal from the measurements. Depending on the spectral transfer function of the filter, an undesirable effect can be induced in the acquired measurements, for example if there are oscillations in the spectral transfer function. Having a good spectral resolution with a great sensitivity in the correct spectral bandwidth is a crucial point to perform the correct measurements, which describe precisely the spectral shape of the fluorescent signal (Haj-Hosseini et al., 2010; Kim et al., 2010; Valdés et al., 2011c, 2012c; Stummer et al., 2014; Stummer, 2015; Alston et al., 2019).

For all fluorescence spectroscopy systems, how excitation of fluorophore is performed influences the final characteristics and performance of the system. Indeed, choice of the number and type of excitation sources [light emitting diodes (LED), laser diode, spectrally filtered broadband source] is crucial to the spectral selectivity of fluorescence excitation. Indeed, LEDs have a higher spectral full width half maximum (FWMH) than laser diodes, which in the case of several spectrally closed excitation sources can lead to a loss of selectivity in fluorophore excitation (Gautheron et al., 2023a).

Then, the spectrally-resolved data are processed using spectral unmixing algorithms (Toms et al., 2005; Ando et al., 2011; Bravo et al., 2017; Zanello et al., 2017; Black et al., 2021). Indeed, signals from several endogenous fluorophores are present in the global signal acquired by the fluorescence intensity spectroscopy system. However, the diagnostic power of fluorescence spectroscopy relies on its ability to separate the PpIX signal from that of other fluorophores. Spectral unmixing of the PpIX signal is required to distinguish the PpIX specific fluorescence signal from the non-specific, global fluorescence signal from other fluorophores such as nicotinamide adenine dinucleotide (NADH), flavin adenine dinucleotide (FAD), flavins or lipopigments. Thus, spectral unmixing is a method for resolving crosstalk between the fluorescence emission spectra of several fluorophores (Kaneko et al., 2019; Black et al., 2021). This methodology assumes that the signal intensity of each wavelength in the acquired spectrum can be presented as a linear combination of fluorescence spectra of several substances whose spectra are known or unknown. The signals of components which are not PpIX can either be assumed to be known (Montcel et al., 2013), assumed to have a specific signal shape (such as a Gaussian; Dietel et al., 1997; Zanello et al., 2017; Alston et al., 2019), or estimated through blind unmixing routines (Haj-Hosseini et al., 2010; Haidar et al., 2015; Leclerc et al., 2020). Further, another difficult aspect of spectral unmixing methods is that photoproducts may appear in the acquired signal as a result of photobleaching or chemical rearrangement following fluorescence excitation.

In addition, recent work has demonstrated that taking into account the two proposed PpIX spectral-states improves detection of LGG and regions of infiltrating gliomas (Montcel et al., 2013; Alston et al., 2018, 2019). More specifically, the Lyon group identified a second spectral-state of interest with a major peak at 620 nm, called PpIX620, which improves the separation between healthy tissue and infiltrated tissue containing lower tumor cell concentration (Alston et al., 2019). This spectral-state is likely attributed to a different aggregate form of PpIX, compared to the main aggregate called PpIX634 with a major peak at 634 nm (Montcel et al., 2013). Other studies suggest attributing a different origin, linking this fluorescence spectrum with a peak at 620 nm to other fluorophores like water soluble porphyrins such as uroporphyrins (Dietel et al., 2007; Zanello et al., 2017). Regardless of the biological origin, which is still open to discussion, but studies have included this second form, PPIX620, in the processing of spectrally-resolved data acquired during 5-ALA-induced PpIX FGS of gliomas, and demonstrated its clinical relevance (Black et al., 2021). However, as the emission spectra of the two proposed PpIX states are spectrally close together by only 14 nm, spectral unmixing can be a challenge. In addition, several spectroscopic studies have exploited tissue autofluorescence or the main PpIX emission peaks from raw fluorescence intensities to demonstrate increased sensitivity for tumor detection (Lin et al., 2001, 2005, 2009, 2010; Toms et al., 2007). Stummer (2015) demonstrated improved diagnostic capabilities over visible fluorescence by using a contact probe spectroscopy system for 5-ALA-induced PpIX resection. Indeed, they found a strong correlation of “strong” and “weak” visible fluorescence qualitative assessments with a stronger and weaker spectroscopic fluorescence intensity signal, respectively.

Next, it is important to distinguish between qualitative and quantitative spectroscopic approaches. All spectroscopic, contact probe technologies approaches have improved light delivery (i.e., illumination) and collection efficiency compared to conventional clinical microscopic approaches. Reflectance and/or fluorescent light is detected by a spectrometer in a spectrally-resolved manner. This light is converted to a “number” of arbitrary units (a.u.) of light intensity at each wavelength. The detected intensity will depend on system characteristics and tissue fluorescent markers (e.g., excitation of PpIX at 405 nm vs. 633 nm) and the tissues optical properties (i.e., absorption, scattering). All these factors will influence the intensity measurements such that two systems can have two completely different intensity measurements of the same tissue. Further, even with the same system, two measurements can yield different intensities depending on such parameters. As such, every assessment of tissue fluorescence is relative or subjective between observers, and can therefore lead to inaccurate estimates of tissue fluorophores given the significant system-based differences (hardware, acquisition parameters) and the non-linear effects due to tissue optical properties. For all these reasons, qualitative methods are not suitable for obtaining absolute fluorophore concentrations and limit the accuracy of comparative studies between different systems.

However, it is possible to achieve quantitative measurements of the fluorescence, i.e., a fluorophore concentration in standardized units, by accounting for system parameters with adequate calibration techniques, and more specifically, by correcting the measured fluorescence signal from the distorting effects of scattering and absorption caused by propagation excitation light and fluorescence emissions in tissue. Spectroscopy systems which couple both fluorescence and diffuse reflectance measurements have been developed (Richards-Kortum and Sevick-Muraca, 1996; Ramanujam, 2000; Kim et al., 2010; Valdés et al., 2011b,c, 2015). DRS data are acquired in a spatially- or time-resolved manner to extract tissue optical properties using post-processing techniques. Tissues are illuminated in a point-wise manner to have access to the tissue point spread function (PSF). The PSF is directly linked to the scattering and absorption properties of the tissue and with use of a fiber optic system, it is possible to sample the PSF at various known and fixed distances from the illumination point. These measurements are then processed by inversion of a light propagation model (see next paragraph) to estimate the optical properties using *a priori* knowledge of the main absorption components (water, oxy-, deoxy-hemoglobin) in tissue. In addition to a more complex acquisition process, this type of DRS measurement implies also a more complex processing (e.g., optimization algorithms for fitting model to data, system calibration). The processing of acquired data requires use of light transport algorithms to correct for tissue attenuation. Attenuation correction techniques can be either empirically-based techniques and theory-based ones. Two comprehensive presentations of fluorescence attenuation correction techniques are noted in Durkin et al. (1994), Richards-Kortum and Sevick-Muraca (1996), Zhadin and Alfano (1998), Mycek and Pogue (2003), Bradley and Thorniley (2006), Tuchin (2015c), and Boas et al. (2016).

Theory-based attenuation correction techniques depend on the considered light propagation assumptions. In cases where the wave nature of light has no significant effect such as diffraction or interferences, which is true for most random media illuminated with a large and broad-spectrum light source, the radiative transfer equation (RTE) can be used to describe the scattering of light within the medium. The non-approximated RTE is an integrodifferential equation whose difficulty of solution lies mainly in the evaluation of the integral term. The radiative transfer approach has been extensively investigated since the 50’s. Following the pioneering work of Chandrasekhar (1960) for the study of the atmosphere (Ishimaru, 1978), the radiative transfer theory can be extended to the propagation of multiple wave types like optical, acoustic, and microwave, and can apply in broader fields such as scattering in atmospheres, oceans, and biological media. The use of an approximate RTE solution under experimental conditions is often due to experimental constraints, e.g., it is not possible to perform an *in vivo* transmittance measurement during neurosurgery. As computer performance improves, inverse approaches using Monte-Carlo (Jacques, 1998, 2003) and symbolic Monte-Carlo methods (Galtier et al., 2017; Maanane et al., 2020) are also being developed. Whatever the model used to solve the RTE, measured fluorescence signal is then corrected using the estimated local optical properties of the tissue. To summarize, one can place probe on the brain tissue, collect spectroscopic data, quantify fluorescence via correction algorithms and ultimately, derive a final output of the quantitative fluorescence estimates of a fluorescent biomarker (absolute PpIX concentration) through the process of fluorescence quantification. Quantification requires (a) collection of both DRS and fluorescence data in a spectrally-resolved manner and spatially-resolved manner; (b) estimation of optical properties; (c) use of optical properties in a model of the fluorescence to correct the raw fluorescence into quantitative fluorescence; and (d) extraction of concentration and contributions of PpIX and other markers of interest using spectral unmixing techniques. For each of these steps, different tools are presented in this article. The choice of tools used depends on geometric and computational constraints, or the publication date of the article.

### 3.2 Time-resolved fluorescence spectroscopy

In addition to fluorescence intensity-based approaches, tools for studying fluorescence lifetime have also been developed for neurosurgery. Fluorescence lifetime techniques, including spectroscopic- and imaging techniques, are able to resolve the different lifetimes of fluorophores, unlike fluorescence intensity approaches which only measure intensity. The fluorescence lifetime of fluorophores is independent of the scattering and absorption of tissues and thus, in essence, quantitative. However, the fluorescence lifetime of fluorophores depends on the microenvironment of molecules. Thus, fluorescence lifetime approaches provide an additional mechanism of optical contrast by taking advantage of differences in the tumor tissue microenvironment on the detected fluorophores. For example, and beyond the scope of this review, the literature on fluorescence lifetime measurements of endogenous markers such as NAD(P)H and flavin in gliomas is abundant, providing an alternative way of evaluating the tissue environment and photoactivity (Stummer et al., 1998b; Yong et al., 2006; Butte et al., 2010, 2011; Stummer et al., 2014; Stummer, 2015; Kaneko and Kaneko, 2016; Kittle et al., 2016; Mehidine et al., 2019; Lukina et al., 2021; Alfonso-Garcia et al., 2023). More recently, articles on autofluorescence lifetime spectroscopy coupled with PpIX lifetime spectroscopy have also been described as a means of fluorescence-based tumor contrast (Mehidine et al., 2019; Erkkilä et al., 2020; Reichert et al., 2021; Alfonso-García et al., 2022).

During the last two decades, point-based fluorescence lifetime spectroscopy instrumentation for tissue diagnosis have been reported, with only a few used for intraoperative measurements of brain tumors (Fang et al., 2004; Butte et al., 2010; Sun et al., 2010; Butte et al., 2011). Time-resolved fluorescence spectroscopy (TRFS) systems share the same requirement of fast recording of spectrally resolved fluorescence decays. For this reason, these systems use a pulsed sampling technique to simultaneously measure the emission spectrum and wavelength-dependent decay characteristics. They are thus composed of a nanosecond pulsed laser for fluorescence excitation delivered via a fiber optic probe. Either the detection part is multispectral using avalanche photodiodes after a band pass filter of different spectral bands (Alfonso-García et al., 2022) or hyperspectral using a dual mode imaging spectrograph coupling a grating with either GHz gated multichannel plate photomultiplier tube (MCP-PMT; Alfonso-García et al., 2022) or an intensified CCD (ICCD) camera (Sun et al., 2010). To synchronize the gating electronics of the detectors (PMT, MCP-PMT or ICCD) and the laser emission, a digital pulse/delay generator is required to generate synchronized pulse trains at variable delays to accommodate for the electronic delays and jitter, as well as the optical delays due to light propagation. A detailed description of TRFS instrumentation can be found in work from the Marcu group (Fang et al., 2004; Marcu and Hartl, 2012). Recent emergence of silicon avalanche photodetectors is also reported, which promise to improve the performance of TRFS systems by increasing imaging speed and reducing lifetime measurement variability by a factor of 5 (Ghezzi et al., 2021; Zhou et al., 2021; Alfonso-García et al., 2022).

In terms of data processing, since several fluorescent molecular species undergo simultaneous excitation, fluorescence intensity decay profiles that result from time-resolved measurements are often complex. Thus, the measured fluorescence decay corresponds to the sum of all individual decays, and the single exponential decay model is therefore inadequate. In practice, in a time-resolved instrument, the response function of the instrument and the laser excitation are generally extended over several nanoseconds. Given these considerations, the observed fluorescence decay, F(t), corresponds to the convolution of the excitation function I_0_(t) with the instrument impulse response function SR(t) and the tissue fluorescence impulse response function IRF(t). Experimentally, we can directly acquire I(t) by direct measurement of laser light, which corresponds to the convolution of SR(t) by I_0_(t). It can therefore be shown that the observed fluorescence decay F(t) is simply the convolution of the measured excitation function I(t) with the tissue fluorescence impulse response function IRF(t). Thus, the intrinsic fluorescence decay can be estimated by deconvolving the observed fluorescence traces F(t) from the excitation pulse I(t), either using an iterative least-squares reconvolution (LSIR) approach (Ware et al., 1973; O’Connor et al., 1979), the Laguerre kernel expansion technique (Jo et al., 2004, 2006) or even via deep learning (Smith J. T. et al., 2020). To visualize the lifetime dynamics, the phasor approach is broadly used (Digman et al., 2008). It plots on a 2D diagram the real and imaginary parts of the Fourier transform of the intensity normalized fluorescence decays.

## 4 Technical considerations regarding current limitations of 5-ALA induced PpIX fluorescence spectroscopy

Here, we present some of the limitations of fluorescence spectroscopy with hand-held point probes. First, we discuss hardware limitations and future developments, highlighting the problems associated with single-point acquisition and the need for spectroscopic imaging approaches. Next, we focus on the limitations of spectral unmixing methods and recent advances addressing these challenges. Finally, we discuss limitations intrinsic to the biology of PpIX as a tumor biomarker and need for improved fluorophores.

The systems presented above perform single-point signal acquisition, which inn practice, has limited utility. Despite very fine spectral resolution and the ease with which any area of the surgical field can be interrogated during resection, the acquisition area remains restricted to a small (~mm) area. Hyperspectral technologies would enable wide-field spectroscopic imaging (Sellar and Boreman, 2005; Valdés et al., 2012a, 2016, 2019; Valdes et al., 2013; Calin et al., 2014; Lu and Fei, 2014; Jermyn et al., 2015; Walke et al., 2023). Thus, these systems perform spectrally-resolved imaging of the (full) surgical field of view, thus enabling visualization of a larger area (~cms) compared to single point spectroscopy systems. Multiple hyperspectral technologies exist including but not limited to snapshot, pushbroom, whiskbroom, and tunable filter approaches. Hyperspectral systems present a promising advancement, having shown in translational and clinical studies improved sensitivity fir detecting tumor tissues previously undetected with conventional commercial microscopes. However, current hyperspectral imaging (HSI) technologies for FGS are associated with technical restrictions that have limited their widespread adoption (Valdés et al., 2012a, 2019; Valdes et al., 2013; Walke et al., 2023). For example, existing systems require a compromise between spectral resolution, acquisition time (Lim and Murukeshan, 2015), spatial resolution, or detection sensitivity (Lu and Fei, 2014). Further, these techniques have been used in empirical approaches (Valdés et al., 2012a; Walke et al., 2023) for quantitative fluorescence, but unlike point spectroscopy approaches, wide field quantitative hyperspectral approaches have not been demonstrated using model based quantitative fluorescence, as these require an additional level of complexity for optical property determination (Cuccia et al., 2009; Valdes et al., 2017; Gioux et al., 2019).

One other main technical aspect limiting the accuracy of 5-ALA induced PpIX fluorescence spectroscopy for surgical guidance is the presence of other non-5-ALA induced fluorophores such as NADH, FAD, flavins or lipo-pigments. The spectral emission variability of these other fluorophores is highly dependent on multiple patient factors including such as aging (Haidar et al., 2015;Alston et al., 2019; Black et al., 2021), and pathology (Ando et al., 2011). In addition to the interpatient variability, there can exist a significant amount of variability between two samples from the same patient (Dietel et al., 2007; Montcel et al., 2013; Zanello et al., 2017;Alston et al., 2019; Black et al., 2021). The presence of fluorophores and their tissue-specific variability (e.g., difference between tumor and normal brain) can lead to important *crosstalk* with PpIX (Black et al., 2021). For example, omitting a fluorophore or using an incorrect basis spectrum will affect the estimated fluorescence signal resulting from PpIX, yielding an over- or under-estimation of PpIX’s amplitude and decreasing the detection accuracy when biomarker contributions are used in a classification pipeline (Alston et al., 2019; Black et al., 2021) between healthy and tumor samples.

To minimize crosstalk, a common approach described in the literature is to model the baseline with every relevant known fluorophore that is not PpIX and are most effective when the emission spectral band of the baseline is far from PpIX (Montcel et al., 2013; Haidar et al., 2015; Black et al., 2021). In these cases, the analytical or empirical *a priori* on the baseline can be a weighted sum of NADH, FAD and flavins spectra (Haidar et al., 2015; Zanello et al., 2017; Black et al., 2021) or an exponential decay function if the wavelength range starts at 500 nm (Ando et al., 2011; Montcel et al., 2013). When the emission spectral band of the baseline is close to or within that for PpIX, as it is the case with lipo-pigments, the existing methods remain limited (Alston et al., 2019; Black et al., 2021). For example, the baseline can be modeled by a Gaussian function (Alston et al., 2019), or an expert-dependent weighted sum, which contains a finite number of fluorophores whose fluorescence spectral shape is assumed to be known (Zanello et al., 2017; Black et al., 2021). However, fluorophores whose spectral emission band overlaps with PpIX are inadequately unmixed with these methods, and include: water-soluble porphyrins (Dietel et al., 1997) with emission spectrum in the 615–625 nm region; or photoproducts produced by photodegradation of PpIX with an emission spectrum in the 620–680 nm region (Dietel et al., 1997; Dysart and Patterson, 2006; Montcel et al., 2013); and lipofuscin (Black et al., 2021) with emissions up to the 620–700 nm region. Another way of solving the crosstalk problem is to estimate the spectral shape of fluorophores using multispectral fluorescence excitation (Gautheron et al., 2023b). This is because the absorption spectrum of different fluorophores does not display the same wavelength-dependent variation. Thus, the combined use of measurements at different excitation wavelengths can improve unmixing, in particular by estimating the signal from endogenous components rather than modeling it. This estimated baseline method assumes that the signal from endogenous components has the same spectral shape at the different excitation wavelengths. These facts highlight the reality that spectroscopy technologies are undergoing exciting developments, and that their current limitations have potential to be overcome to maximize the potential of spectroscopy in neurosurgery.

A final limitation concerns biological considerations, given that the efficacy of PpIX spectroscopy is directly linked to the efficacy of its biomarkers. Indeed, other exogenous biomarkers such as indocyanine green and fluorescein are used in neurosurgery (Pogue et al., 2010; Valdés et al., 2016). Indocyanine green (ICG) is a small, hydrophilic molecule with broad-band absorption and emission spectra, with maximum absorbance at 805 nm and maximum emission around 830 nm. ICG binds to plasma and is thus a marker of vascularization (Ferroli et al., 2011). Although traditionally ICG fluorescence has been described as not tumor-specific (Belykh et al., 2016), recent work on what is called second window ICG, which involve high dose administration of ICG up to 24 h before surgery, has shown the utility of ICG in FGS for identification of high grade gliomas with high accuracy (Cho et al., 2019, 2020; Teng et al., 2021b).

Fluorescein is another marker excited between 460 and 490 nm and emitting light around 510–530 nm. The first use of fluorescein in neurosurgery was in 1948 for localizing brain tumors (Moore et al., 1948; Pogue et al., 2010; Valdés et al., 2015). According to the study of Diaz et al. (2015), fluorescein crosses the leaking blood brain barrier in high-grade gliomas, and thus accumulates in these tumors enabling FGS. In addition, Diaz et al. point out that fluorescein reaches tumor cells through the cerebral vasculature, with no fluorescence detected in the necrotic part of the tumor. These are examples of two other biomarkers which have been used for FGS, however, they are less specific than PpIX. Autofluorescence (Croce et al., 2002; Majumder et al., 2007; Alfonso-García et al., 2022) has also been studied for classifying healthy and tumoral tissues, but has not shown significant utility as a surgical adjunct as has 5-ALA induced PpIX fluorescence (van Beurden et al., 2020).

## 5 Clinical issues

The majority of studies using 5-ALA-induced PpIX fluorescence spectroscopy have been performed in HGG, more specifically, in GBM, since 5-ALA is approved for FGS in this tumor type, has proved to be cost-effective (Picart et al., 2023), and has become a standard of care worldwide (Stummer et al., 1998a; Díez Valle et al., 2011; Panciani et al., 2012; Kim et al., 2014; Li et al., 2014; Chan et al., 2018). However, 5-ALA-induced PpIX fluorescence spectroscopy has also been used in FGS across other different pathologies including LGG, meningiomas and metastases. Here we present an overview of intraoperative implementations of 5-ALA-induced PpIX fluorescence spectroscopy in neurosurgery, describing the various implementations and their added value in clinical practice.

Stummer et al. (1998b) performed, to our knowledge, the first *in vivo*, intraoperative 5-ALA induced PpIX fluorescence spectroscopy measurements in GBM. In this study, the authors detected the typical PpIX spectra and main peaks at 635 and 704 nm in regions with visibly red-pink fluorescent tissue, verifying the presence of PpIX specific fluorescence. Ishihara et al. (2007) used a spectroscopy probe to collect fluorescence emissions and reflected excitation to calculate a fluorescence intensity ratio described as the ratio of fluorescence emissions intensity over reflected excitation intensity. They found that a ratio of <0.001 corresponded to normal tissues and a ratio > 0.090 corresponded to glioblastoma (GBM) tissues (Ishihara et al., 2007). Utsuki et al. (2008) analyzed data on six patients with HGG following 5-ALA administration and using a fluorescence spectroscopy probe. They found that a difference in fluorescence intensity of >500 in arbitrary units of fluorescence intensity between the intensity at 632 and 636 nm, following 405 nm excitation, could detect PpIX fluorescence that was otherwise invisible to the naked eye in 3 out of 6 patients, in histologically confirmed tumor infiltrated tissues.

In another study on GBM, Haj-Hosseini et al. (2010) used a fluorescence spectroscopy system with pulsed modulation to minimize photobleaching and omit ambient light on patients undergoing FGS with 5-ALA. The system performed 405 nm excitation with collection of fluorescence intensity peaks at 635 and 704 nm and autofluorescence at 510 nm. Then they used a ratio of the PpIX peak to the autofluorescence for tissue discrimination. This system also used the intensity at different peaks with arbitrary units of intensity and a fluorescence ratio calculation. However, this system was unique in its use of pulsed fluorescence spectroscopy to suppress the low power ambient lighting and enable improved detection of GBM tissue. In a follow up study by this same group, Richter et al. (2011) used a fluorescence spectroscopy probe combined with an ultrasonic navigation system to couple detection with navigated resection on nine patients with GBM following 5-ALA administration. They used the same ratio of the fluorescence intensity at 635 nm to the autofluorescence for classification of tumor from non-tumor tissue and found that they could differentiate with high statistical significance of *p* < 0.001. They found a median fluorescence ratio in non-tumor tissue of 0, low (0.63) in gliotic or infiltrative zones, and highest in the solid tumor (2.52) in over 180 points. This system also implemented a ratio-based metric for differentiation of tumor from non-tumor tissue, but was unique in its coupling with ultrasonic navigation. Richter et al. (2017) then tested this same system on 16 patients with GBM and performed one to one comparisons of their spectroscopic fluorescence ratio approach to that of conventional visible fluorescence assessments using a microscope. The authors found that up to 67% of non-visibly fluorescent tissue demonstrated significant PpIX fluorescence with the probe. Further, diagnostic performance studies using receiver operating characteristic (ROC) curve analyses, found the area under the curve (AUC) of the spectroscopy system was 0.65 and that of the microscope was 0.49, with different sensitivity and specificity values for the probe based on the ratio used. This work provided improved accuracy for tumor detection using a spectroscopy system and a ratio-based approach compared to conventional fluorescence microscopy. However, results for fluorescence microscope were lower than those reported in previous spectroscopy studies by other groups. Haj-Hosseini et al. (2015) used their spectroscopic system to determine if there was a difference in fluorescence detection in 30 patients with HGG receiving either standard, high doses of 5-ALA at 20 mg/kg (*n* = 15) vs. low doses at 5 mg/kg (*n* = 15). They found that median fluorescence ratio and PpIX fluorescence was 2–3 times greater in the high 5-ALA dose group. However, there was no significant difference in diagnostic performance between the low and high 5-ALA groups. This work demonstrated the utility of more sensitive measurements afforded by spectroscopy in determining any significant differences between drug doses and fluorophore levels. Most studies to date using fluorescence spectroscopy have been in adults. However, Milos et al. (2023) used the spectroscopy system by Haj-Hosseini et al. (2010) on 13 pediatric patients with a variety of pediatric brain tumors including medulloblastoma, GBM, pilocytic astrocytoma, meningioma and diffuse intrinsic pontine glioma. Similar to before, the authors used the ratio-based approach and found improved detection of PpIX fluorescence and tumor tissue compared to conventional fluorescence microscopy. Specifically, microscopy only identified visible fluorescence in two tumors meanwhile spectroscopy detected PpIX fluorescence spectra in five tumors.

Kairdolf et al. (2016) developed a similar fluorescence spectroscopy probe consisting of a single central fiber for emission collection surrounded by six fibers for excitation at 405 nm, and used fluorescence intensity in arbitrary units as the differentiation metric. They found in *ex vivo* human GBM tissues that tumor generally displayed over 100 times more fluorescence than background signals in non-GBM tissues. Cornelius et al. (2017) performed *ex vivo* measurements of GBM (6) and meningiomas (5) using both a clinical spectroscopy probe with reported similar fiber arrangements as that in Kim et al. (2010) and Valdés et al. (2011b) and a laboratory benchtop spectrometer. They found that the clinical probe could detect fluorescence intensity with high sensitivity in both contact and non-contact mode, and as expected, the non-contact mode demonstrated decreasing fluorescence intensity with distance from the sample (Cornelius et al., 2017). Reichert et al. (2021) performed co-validation of their novel fluorescence lifetime imaging system with fluorescence spectroscopy on over 150 specimens from LGG, HGG, meningiomas and metastases. The authors used the relative signal contribution of PpIX at 635 nm as the metric for classification, which involved a ratio of PpIX fluorescence intensity at 635 nm and autofluorescence intensity. This study was unique in performing co-registered studies between PpIX fluorescence lifetime and PpIX fluorescence spectra and ratios.

Montcel et al. (2013) have demonstrated the importance of multiple PpIX photochemical states during 5-ALA induced PpIX FGS, unlike all other previously described methods focused on the main 634 nm peak. The Lyon group developed a spectroscopy method which measures the two peak emissions of PpIX at 620 and 634 nm, and by using the ratio of the fluorescence intensities at 620/634, they were able to more accurately distinguish the infiltrative GBM zones and LGG tissues compared to a single peak approach in four patients. Previous results were confirmed in an *in vivo* study on 10 patients, which demonstrated better separation of healthy tissue and tumor infiltration using the two emission peaks (Alston et al., 2019). The Lyon group used a spectroscopy system in a follow up study in Leclerc et al. (2020) to implement a machine learning approach for improved margin detection in 5-ALA FGS. They investigated 50 samples from 10 patients with glioma, and implemented a feature search to select the best features to achieve an optimal accuracy of 77%. It is important to note that this work focused on margin detection which is a more challenging problem than distinguishing tumor core from normal tissue.

Valdés et al. (2011b) used a spectroscopy probe that enabled quantitative estimates of PpIX concentrations by collecting both fluorescence emissions and white light reflected light, unlike all prior methods using either arbitrary units of intensity or intensity ratios. The authors use this data to estimate the tissue optical properties using a spatially-resolved model of the diffuse reflectance. Then, the authors use this estimate as input in a light transport model to correct the raw fluorescence, estimate the corrected absolute fluorescence, and use spectral unmixing techniques to extract the absolute concentration of PpIX in tissues. They used this system across multiple tumor types including HGG, LGG, metastases, and meningiomas. They found that quantitative fluorescence with PpIX concentration as the metric for differentiation increased accuracy to 87% compared to conventional microscopy assessments of the visible fluorescence with an accuracy of 66%. Furthermore, they found that accuracy for LGG using the probe was similar to that of visible fluorescence for HGG. This finding was notable, since previously it was thought that PpIX cannot be used to distinguish tumor tissue in LGG. In a follow up study (Valdés et al., 2015) on 12 patients with LGG, the authors confirmed these findings using ROC analyses, with an AUC of 0.514 and accuracy of 38% for visible fluorescence and AUC of 0.66 and accuracy of 67% for the quantitative fluorescence approach using PpIX concentrations as the differentiating metric. The authors found that up to 45% of non-visibly fluorescent confirmed tumor samples were accurately detected using a PpIX concentration metric with a threshold of >0.006 ug/mL. A follow up collaborative study using the same system at a different institution and in 22 patients with LGG, Widhalm et al. (2019) showed reproducible, consistent results to those of the original Valdes et al. experience with LGG, demonstrating an increased AUC, sensitivity, negative predictive value, and accuracy as well as detection of 40% of non-visibly fluorescent tumor positive samples using quantitative PpIX fluorescence compared to conventional visible fluorescence microscopy.

Valdés et al. (2011b) implemented a machine learning approach using a support vector machine (SVM) classification algorithm on 10 gliomas (HGG, LGG, and recurrent glioma) and over 264 data points (88 sampled sites) with the quantitative probe results. In this work, the authors used multiple quantitative biomarkers as input to the SVM algorithm, which were derived from their model-based approach, including PpIX concentration, hemoglobin concentration, oxygen saturation and scattering parameters. They found that their accuracy increased to 94% (AUC of 0.94) compared to use of PpIX concentration alone with an accuracy of 83% (AUC of 0.87) or visible fluorescence with an accuracy of 64% (AUC of 0.72). This work demonstrated the potential of quantitative assessments of fluorescence coupled with multiple additional quantitative metrics as one of the first implementations of machine learning algorithms in fluorescence spectroscopy during FGS of brain tumors. Valdés et al. (2012c) used this same system to develop and validate an empiric fluorescence correction algorithm, by first validating in phantoms, and ultimately testing real life human spectroscopy data. They found that their empiric correction algorithm enabled accurate estimation of PpIX concentrations with R^2^ = 0.9942 in phantoms, and an AUC of 0.92 in *in vivo* human data acquired in 14 patients undergoing resection.

As noted above, the most common pathologies investigated with 5-ALA fluorescence intensity spectroscopy have been gliomas. However, other pathologies such as meningiomas and metastases have been explored. Valdes et al. (2014) described one of the largest experiences with meningiomas in 10 patients and 49 tissue sites using their quantitative probe. They found that 39% of tumor specimens did not display visible fluorescence, however, 69 of these contained significant PpIX concentrations as determined by spectroscopy. Also, quantitative fluorescence had a significantly (*p* < 0.007) greater accuracy of 84% compared to conventional visible fluorescence microscopy of 71%. The same group also described the use of quantitative fluorescence spectroscopy in a skull base meningioma, with improved detection in areas invisible to the surgical microscope (Bekelis et al., 2011). Other studies on meningiomas include Haj-Hosseini et al. (2010) in pediatric meningiomas; Reichert et al. (2021) on 18 meningioma samples in a combined lifetime and intensity study; and Cornelius et al. (2017) in five meningiomas *ex vivo* with reported greater fluorescence intensities in all GBM samples compared to meningiomas.

Similar to meningiomas, the experience with metastases is also limited. Reichert et al. (2021) on 35 metastases samples in a study focused on lifetime validation with fluorescence intensity. Valdés et al. (2011b) reported a small experience on 12 samples from 3 metastases patients using their quantitative probe. They found, similar to their experience with HGG, LGG, and meningiomas, improved accuracy using quantitative fluorescence spectroscopy with AUC of 0.98 for PpIX concentration compared to 0.75 for conventional visible fluorescence microscopy.

Finally, although the focus of this review centers on 5-ALA-PpIX, which is the standard of care fluorescent agent for FGS in HGG, it is important to note fluorescein sodium (FS) and indocyanine green (ICG) have also been employed as agents for use in FGS (Pogue et al., 2010; Zehri et al., 2014; Senders et al., 2017). FS was the first fluorescent agent used for FGS in 1948 by Moore et al. (1948) and it is a well-known agent used in other areas of medicine/surgery including ophthalmology and neurosurgery (e.g., cerebrospinal fluid leak evaluation; Pogue et al., 2010; Senders et al., 2017). FS is administered intravenously and is excited at 460 nm, emitting fluorescence at 515 nm of the visible spectrum. Unlike PpIX which has demonstrated rather high specificity for tumor tissues, FS is a non-specific tumor agent, ultimately accumulating in tumors and vasculature alike via the enhanced permeability and retention effect(s); as such it appears to accumulate in brain tumors only if they demonstrate a compromised blood brain barrier (BBB; Pogue et al., 2010; Senders et al., 2017). As such, care must be taken when using FS, as it can accumulate in other areas in which the BBB and/or vasculature are disrupted regardless of the presence of tumor tissue (Hansen et al., 2019; Stummer et al., 2019). However, with knowledge of its limitations and possible false positives, it can nonetheless serve as a useful surgical adjunct (Hansen et al., 2019; Suero Molina and Brokinkel, 2020).

ICG is a NIR fluorescent agent that is excited in the 760–800 nm range, emits NIR fluorescence light >800 nm and is commonly used for aneurysm and arteriovenous malformation surgeries (Raabe et al., 2005; Zehri et al., 2014; Foster et al., 2019). Similar to FS, ICG accumulates in tumors via the enhanced permeability and retention effects as it binds albumin in the vasculature and as such, demarcates vasculature within tumors (Hansen et al., 1993; Martirosyan et al., 2011; Zehri et al., 2014). Interestingly, a substantial body of literature also exists which has described the second window ICG (SWIG) phenomenon, in which ICG is administered up to 24 h prior to surgery, leads to a high tumor-to-normal fluorescence contrast (Teng et al., 2021a,b).

One major advantage of ICG as compared to 5-ALA-PpIX or FS is that it functions as a NIR fluorescent agent, and thus, is less affected by autofluorescence and significant hemoglobin absorption encountered in the visible range of the spectrum, enabling greater depth of penetration (i.e., up to several millimeters; Pogue et al., 2010; Teng et al., 2021b). However, given its NIR emission, it is not visible to the naked eye through the surgical oculars or with conventional color cameras, and therefore requires cameras/optical systems with high efficiency in the NIR range as well as digital image processing for image overlays to visualize the ICG NIR fluorescence in the setting of the underlying anatomy.

## 6 Conclusion and future directions

FGS has been shown to increase the extent of resection, which is of paramount importance given that maximally safe resection of GBM improves progression-free survival and overall survival. Maximally safe resection is also associated with positive symptom management and health-related quality of life, independent of age, clinical status, and adjuvant oncological treatments (Lacroix et al., 2001; Sanai and Berger, 2008; Sanai et al., 2011a; Grabowski et al., 2014; Li et al., 2016; Picart et al., 2023). However, conventional surgical microscopes modified for 5-ALA-PpIX FGS have limited sensitivity and specificity. As described above, spectrally-resolved detection using handheld spectroscopy devices has been shown to significantly improve the diagnostic capability of 5-ALA PpIX for FGS. Thus, technologies with improved diagnostic capabilities and imaging performance are poised to maximize the targeting potential of 5-ALA-PpIX as a tumor biomarker (Valdés et al., 2016, 2019). As noted above, HSI has been recently implemented in FGS using 5-ALA PpIX as a means to perform spectrally-resolved detection across the full surgical field of view to harness the improved diagnostic potential provided by spectroscopic detection (Valdés et al., 2012a, 2016; Valdes et al., 2013; Lu and Fei, 2014; Jermyn et al., 2015; Xie et al., 2017; Kaneko et al., 2019; Schwake et al., 2019; Walke et al., 2023). HSI allows the user to perform “fluorescence guided imaging spectroscopy” rather than single point spectral detection done with handheld spectroscopy devices (Lu and Fei, 2014; Valdés et al., 2019). Valdés et al. (2012a) were the first to report on the development of a liquid crystal tunable filter (LCTF)-based HSI clinical system for use in glioma surgery. In their translational work which included system design, development, validation, pre-clinical animal studies, and ultimately human implementation, they demonstrated improved detection abilities of HSI for 5-ALA-PpIX. They showed that their HSI system could detect tumor tissues in both animals and in human GBM that were not detected (i.e., “invisible”) with current commercial systems (e.g., Zeiss fluorescence enabled microscope). This group subsequently developed improved versions of this LCTF-based HSI system that demonstrated improved sensitivity and acquisition speeds to help improve the diagnostic capabilities of 5-ALA-PpIX (Valdes et al., 2013; Jermyn et al., 2015). Kaneko et al. (2019) implemented this LCTF-based technology for HSI in a cohort of 68 patients for *ex vivo* tissue analysis to enable real-time kinetics measurements of PpIX and found that PpIX fluorescence is maximal at later points than previously described in the literature. This same group used has also used this LCTF-based HSI system for PpIX detection in pediatric tumors (Schwake et al., 2019), and has demonstrated more sensitive detection of autofluorescence and PpIX spectral features such as the 620 and 634 nm states in *ex vivo* samples from 128 patients with HGG and LGG (Black et al., 2021), and implemented new phantom recommendations for HSI in 5-ALA-PpIX FGS (Walke et al., 2023). Another use of HSI that is being explored by an ongoing European collaborative is centered on a system that may ultimately be capable of mapping, monitoring and quantifying biomolecules of interest; it has been engineered to be handheld and user-friendly, and apply artificial intelligence-based methods for the reconstruction of spectrally-resolved images (Giannoni et al., 2023). Such efforts are positioning HSI technologies in a way that may potentially improve diagnostic performance across the full surgical field of view in 5-ALA-PpIX FGS.

In addition to technological advances that seek to improve detection, future investigation is needed to determine how advanced spectroscopic methods might enable prediction of the molecular profile of tumors, and how those profiles might influence intraoperative fluorescence. For example, studies have found a significant positive correlation between World Health Organization (WHO) grade and Ki-67/MIB index (Valdés et al., 2011a; Jaber et al., 2016), but not with *MGMT* promoter methylation, IDH1 mutation status, or *1p19q* co-deletion status. However, a study by Saito et al. looking at 60 patients, found that IDH1 status was the only independent and statistically significant factor predictive of intraoperative fluorescence (Saito et al., 2017). Gene expression patterns from 14 GBM patients found that non-fluorescent tumors tended to resemble the neural subtype of GBM (Almiron Bonnin et al., 2019). Others found PpIX fluorescence as a useful imaging marker to help isolate invasive GBM cells to enable subsequent RNAseq studies (Smith S. J. et al., 2020). Further, another future use of 5-ALA FGS is the implementation of fluorescence-guided biopsies, which are being used in different neurosurgical centers, given their improved diagnostic yields as compared to typical frozen sections (Shofty et al., 2019; Malinova et al., 2020; Millesi et al., 2020).

In summary, the use of fluorescence spectroscopy in clinical practice during 5-ALA FGS dates back >25 years, with the first reported case by Stummer and colleagues, confirming the spectral signature of PpIX in the visibly red-pink fluorescence tissue observed during their first HGG surgeries with 5-ALA. Since then, various groups across the world have implemented fluorescence spectroscopy to improve the detection of tumor tissue and thus, enable more accurate fluorescence guidance during brain tumor surgery. Most studies to date have been on GBM, most likely since 5-ALA is approved for use in HGG. However, additional pathologies such as LGG, meningiomas, and metastases have shown promising results. Different clinical systems and approaches have been implemented, with the most common relying on some form of fluorescence intensity or ratios of fluorescence intensity as metrics for differentiation of tumor from normal tissues. These methods all use arbitrary units of fluorescence intensity and as such, are ultimately prone to subjectivity and relative in their measurements. Recently, a quantitative spectroscopy approach that uses a light transport model to correct the fluorescence for the effects of tissue optical properties was developed with a large experience both from the original group and by collaborating groups, demonstrating the reproducibility and utility of this approach to enable absolute measurements of PpIX concentrations. Further, HSI-approaches which enable “imaging spectroscopy” for 5-ALA-PpIX in the clinical setting have shown improved detection capabilities for tumor tissue beyond conventional commercially available clinical systems. Finally, all these spectroscopic approaches, regardless of the technical differences, demonstrate improved detection of PpIX fluorescence across tumor types, allowing the user to detect tumor tissues that would have otherwise gone undetected with conventional fluorescence microscopy approaches.

## Author contributions

AG: Writing – original draft, Writing – review & editing. JB: Writing – review & editing. TP: Writing – review & editing. JG: Writing – review & editing. PV: Writing – original draft, Writing – review & editing. BM: Writing – original draft, Writing – review & editing.
